# Clear Aligners as a Prescribed Source of Unmeasured Intra-Oral Micro- and Nanoplastic Exposure: Release Mechanisms, Human Evidence, Salivary Biomonitoring and the EU Regulatory Context

**DOI:** 10.3390/ma19183816

**Published:** 2026-09-08

**Authors:** Marcin Mikulewicz, Katarzyna Chojnacka

**Affiliations:** 1 Division of Facial Abnormalities, Department of Dentofacial Orthopaedics and Orthodontics, Wroclaw Medical University, Krakowska 26, 50-425 Wroclaw, Poland; 2 Department of Advanced Material Technologies, Faculty of Chemistry, Wroclaw University of Science and Technology, Wybrzeze Wyspianskiego 27, 50-370 Wroclaw, Poland; katarzyna.chojnacka@pwr.edu.pl

**Keywords:** clear aligners, dental polymers, microplastics, nanoplastics, antimony, salivary biomonitoring, safe-and-sustainable-by-design

## Abstract

**Highlights:**

- Aligners shed micro- and nanoplastics in vitro; antimony release under gastric acid (one study).
- Sub-5 micrometre and nanometre fractions are the most plausible translocation candidates.
- Patient internal dose is unmeasured; saliva is a tractable biomonitoring matrix.
- EU amalgam phase-out highlights, but does not directly cause, a broader polymer-substitution risk.
- A harmonised leaching protocol and salivary biomonitoring are proposed, with children prioritised.

**Abstract:**

Clear orthodontic aligners are removable polymer devices worn about 22 h a day for 12 to 24 months, increasingly by children. A growing in vitro literature shows that thermoformed and 3D-printed aligners shed micro- and nanoplastics and co-release elemental or organic constituents under oral, gastric and mechanical conditions, yet aligner-derived particle burden and systemic dose in wearers have not been measured. This review sets aligner-derived release against the wider human micro- and nanoplastic record and evaluates saliva as a biomonitoring matrix. Directly printed devices release more and larger microplastics under mechanical stress, whereas thermoformed materials show acid-related degradation, with Sb/Sn release and nanometre-scale particles reported in a single, not yet replicated in vitro study. Particles below 5 microns, especially below 1 micron, are plausible translocation candidates, but translocation of aligner-derived particles has not been demonstrated. In vitro work reports macrophage and oxidative responses, whereas detections in blood, placenta and atheroma are not source-specific and remain associative. The EU amalgam phase-out does not regulate aligners but highlights a substitution question, because device and chemicals law addresses intentionally added microplastics, not wear-generated secondary particles. ICP-MS for antimony and tin, with Raman, micro-FTIR and electron microscopy, forms a plausible analytical toolbox. We propose harmonised release testing and validated salivary biomonitoring, prioritising children, to characterise source-proximal exposure before use expands.

## 1. Introduction

Micro- and nanoplastics (MNPs) have moved from an environmental curiosity to a recognised question of human health. A recent synthesis in *Nature Medicine* concluded that particles may cross epithelial barriers and reach the circulation, and that early clinical signals point to immune, reproductive and cardiovascular effects, while stressing that the human evidence base is not yet reliable enough to support formal risk assessment [1]. Most of that literature concerns diffuse, involuntary exposure through food, water and inhaled air. One exposure that is neither diffuse nor involuntary has received far less attention from the systemic MNP field, even as its own dental literature expands quickly: clear orthodontic aligners [2,3]. Within dental materials science, aligners are a fast-growing polymer device class, and their in-service degradation is a materials-engineering question as much as a clinical one, which is the perspective this review adopts.

Terminology in this field is not fully standardised, and precision matters for what follows. Microplastics are conventionally defined as polymer particles smaller than 5 mm. For the smaller fractions, which carry the biological argument of this review, we apply a four-tier classification and use it consistently throughout: nanoplastics in the strict, ISO-aligned sense (<100 nm); submicron particles (100 nm–1 µm); fine microplastics (1–5 µm); and larger microplastics (>5 µm). Much of the aligner-release literature labels the entire <1 µm range “nanoplastics”. When a cited study does so, we retain its reported numerical range but describe particles of 100 nm–1 µm as submicron rather than nano, reserving “nanoplastic” for <100 nm. The distinction matters because the biological arguments are size-specific: epithelial translocation is most plausible for the <100 nm tier, remains plausible for the 100 nm–1 µm tier, becomes conditional at 1–5 µm and is unlikely for larger fractions, whereas macrophage uptake is documented for particles of roughly 2–10 µm. Across this review, the emphasis therefore lies on the three tiers below 5 µm, since these are the sizes that release studies increasingly report and that translocation studies identify as most relevant.

This is a narrative, integrative review, not a systematic review. The choice of genre is deliberate: systematic and scoping syntheses of the aligner-release literature already exist [4,5] and are compared with the present work above, whereas the question addressed here spans four distinct literatures—polymer materials science, human micro- and nanoplastic evidence, analytical and biomonitoring methodology, and EU device and chemicals regulation—which cannot be collapsed into the single focused question that a systematic review requires. Reporting, therefore, follows the standard appropriate to the narrative genre, the Scale for the Assessment of Narrative Review Articles (SANRA) [6], rather than PRISMA. The evidence base was nonetheless assembled in a structured way. Scopus served as the primary database, supplemented by targeted PubMed/MEDLINE searches and, for legal instruments and standards, EUR-Lex and the ISO catalogue. Searches covered January 2014 to July 2026, when the final search was run, and combined four thematic blocks, adapted to each database’s syntax: (i) device and material terms (“clear aligner*” OR “orthodontic aligner*” OR “dental polymer*” OR PETG OR “thermoplastic polyurethane” OR “3D print*” OR “photopolymer resin”); (ii) release and exposure terms (microplastic* OR nanoplastic* OR “particle release” OR leach* OR antimony OR tin OR “residual monomer*”); (iii) biomonitoring and analytical terms (saliva* OR biomonitor* OR “ICP-MS” OR Raman OR FTIR OR “electron microscopy”); and (iv) regulatory terms (REACH OR “Medical Device Regulation” OR amalgam OR “dental mercury”). Blocks were combined pairwise (i AND ii; ii AND iii; i AND iv; iii with saliva-specific terms alone), and the reference lists of the retrieved reviews [2,3,4,5] were additionally hand-searched. Records were included if they were peer-reviewed publications in English reporting (a) particle or chemical release from clear aligners or related intra-oral polymers, (b) micro- and nanoplastic detection or effects in human matrices or human-relevant models, (c) analytical methods applicable to salivary biomonitoring, or (d) EU regulation of dental materials or microplastics; records were excluded if they addressed only orthodontic efficacy, environmental compartments without human relevance, or were available only as abstracts without extractable data. Both authors screened titles and abstracts against these criteria and assessed candidate full texts, with disagreements resolved by discussion. Scopus AI outputs and bibliographic exports were used only to identify candidate records and themes and were not treated as scientific evidence. Consistent with SANRA, no PRISMA-style flow count is reported: study-level tallies for the aligner-release literature are provided by the systematic and scoping reviews cited above [4,5], whereas the contribution of this review is integrative, and sources are cited selectively for the evidence they add to each of the four strands rather than exhaustively; the review is organised around exposure rather than clinical outcome. Primary legal instruments and relevant ISO standards are cited in full in the References [7,8,9,10,11,12,13,14,15,16,17].

Aligners differ from environmental sources in ways that matter for exposure science. They are prescribed by a clinician, made from defined polymers, worn against warm saliva for the greater part of each day, and replaced on a fixed schedule. That combination makes them a rare instance of an intentional, quantifiable and repeatable oral contact with plastic, sustained over months to years. It also makes them tractable: the device, the dose schedule and the exposed population are all knowable in advance, which is seldom true of ambient exposure. A growing experimental literature now documents both particle and chemical release from these devices, and two independent reviews conclude that release is supported by the current in vitro data while the evidence remains insufficient to characterise human exposure or risk [4,5].

Three recent syntheses cover adjacent ground, and the position of the present review relative to them should be stated explicitly. De Stefano et al. [4] systematically reviewed eleven in vitro studies identified up to December 2024, with objectives confined to particle release and surface change; they concluded that aligner surfaces roughen with use and disperse microplastics of roughly 5–20 µm and closed with a general call for further research and alternative materials. Varghese et al. [5] mapped the literature up to February 2025 in a scoping review of six studies whose stated aims were evidence mapping, health implications and policy directions; they concluded that release during use is documented, but that the magnitude of any health risk is unknown, and that in vivo research and safer materials are required. Umrai Shariff et al. [2] surveyed micro- and nanoplastics across clinical dentistry and orthodontics in a broader narrative review spanning aligners, retainers and adhesives and identified the quantification of appliance-derived leakage and its long-term effects as an open gap. All three reviews therefore converge on the same endpoint: in vitro release occurs, human exposure data are absent, and further research is needed. The present review is designed to begin where those reviews stop. It differs in objective because it does not re-catalogue the release evidence but asks how the missing patient-level exposure can actually be measured; in coverage, because it integrates four strands that none of the cited reviews treats together—particle release alongside co-released elemental and organic chemistry (Sb/Sn, residual monomers), the wider human MNP record and its interpretive limits, the analytical readiness of saliva as a biomonitoring matrix, and the EU regulatory context (the amalgam phase-out, the REACH restriction on synthetic polymer microparticles and the Medical Device Regulation); and in output, because it ends not with a call for further research but with a specified measurement programme: a harmonised in vitro leaching protocol with defined size-resolved read-outs and mandatory procedural blanks, as well as a validated, child-prioritised salivary-biomonitoring framework with named target analytes, candidate instrumentation, QA/QC requirements and identified reference-material gaps. To our knowledge, no previous publication has proposed an operational salivary-biomonitoring framework for aligner-derived micro- and nanoplastic and elemental exposure; that framework, and the regulatory analysis that motivates it, are the intended contributions of this article.

What is missing sits at the centre of any exposure assessment: the internal dose received by the patient. Detection of MNPs in human blood and in carotid atheroma, where particle burden was associated with subsequent cardiovascular events, shows that internal compartments are reachable and that the question is not merely academic [18,19]. Those findings are associative, and they say nothing directly about aligners. They do raise the cost of leaving a prescribed oral exposure unmeasured, particularly as children and adolescents are among the wearers, a group in whom any persistent exposure could accumulate against a longer remaining lifespan and a still-developing physiology [20].

**Figure 1:**
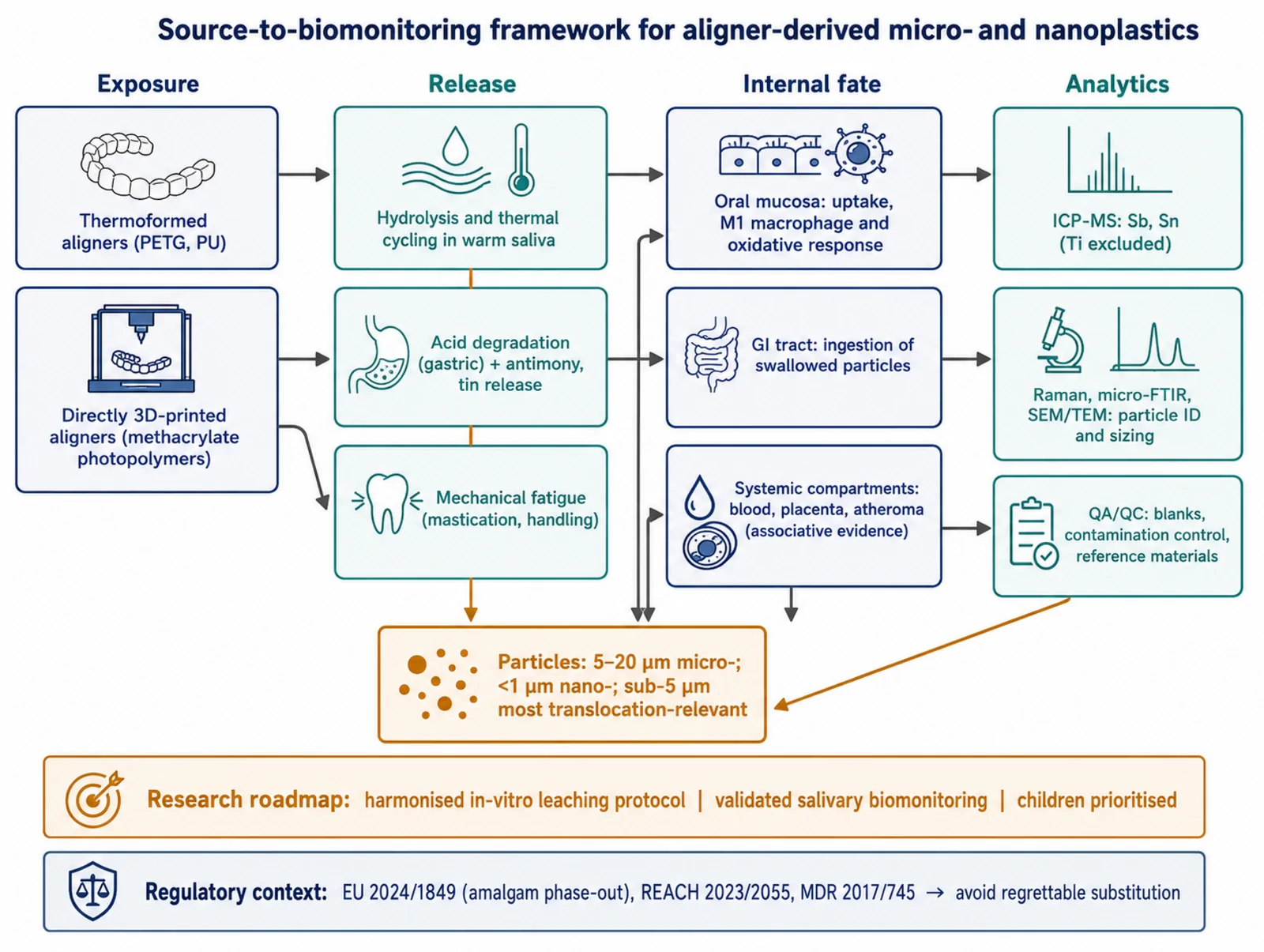
Source-to-biomonitoring framework separating directly observed in vitro release from unverified patient translocation and non-source-specific human detections. The analytical strategy combines elemental and particle methods with strict QA/QC, and the research roadmap prioritises harmonised release testing and validated saliva studies. The pathway is staged as device release → salivary concentration → swallowed/oral-contact dose → epithelial uptake → systemic burden (Table 1); direct evidence currently exists only for the first stage, and the second is the measurement target proposed here.

This review brings together three strands that are usually discussed separately. The first is the mechanism and magnitude of aligner-derived release, covering both particles and the elemental and organic chemistry that degradation liberates. The second is the wider human evidence for MNP internalisation, which sets the biological stakes without over-reaching into causation. The third is the analytical question of how to measure the missing dose, and specifically whether saliva can serve as a first-line, non-invasive biomonitoring matrix. We close with a research roadmap built around a harmonised in vitro leaching protocol and validated salivary biomonitoring, with children prioritised, and we place the whole discussion within the European regulatory shift away from dental amalgam and towards polymers. Figure 1 maps this structure from source through fate and analysis to the regulatory response.

To keep these stages distinct, the review uses a five-stage exposure pathway throughout: (i) device release, established in vitro (Section 3); (ii) salivary concentration in wearers, the source-proximal external exposure this review proposes to measure; (iii) the swallowed and oral-contact dose presented to the gastrointestinal tract and oral mucosa; (iv) epithelial uptake, for which the evidence is mechanistic and largely drawn from model particles (Section 5 and Section 6); and (v) systemic burden, where human detections exist but are not source-specific (Section 7). Evidence for one stage is never treated as evidence for a later one: laboratory release does not demonstrate patient exposure, and human detections do not demonstrate aligner origin. Table 1 summarises the five stages, how each could be measured and the evidence currently available for each.

The stance taken here is deliberately one of measurement rather than alarm. A precautionary reading of an unmeasured exposure can slide into overstatement, while a dismissive reading can slide into unwarranted reassurance, and both fail the same test: they substitute a prior for a measurement. A defensible safety assessment should therefore include measurement of what the device releases and of the exposure experienced by the patient, without presuming either harm or absence of harm.

The scope of this review is exposure, not orthodontic performance. Aligners are effective, patient-preferred appliances, and nothing here questions their clinical value or advocates a return to fixed appliances; the aesthetic and functional case for aligner therapy is well established and lies outside our subject. The narrow claim is that a device delivering prolonged intra-oral polymer contact warrants exposure characterisation appropriate to materials placed in sustained contact with oral tissues, and that particle exposure characterisation is currently missing. Framing the question this way separates a legitimate materials-safety inquiry from any implication that the treatment itself is in doubt.

## 2. Aligner Materials, Manufacturing and Clinical Use

Two manufacturing routes dominate the market and shape every downstream exposure question. Thermoformed aligners are produced by pressing a heated polymer sheet, most often polyethylene terephthalate glycol or thermoplastic polyurethane, over a physical or printed dental model, then trimming the result. Directly 3D-printed aligners are built layer by layer from methacrylate-based photopolymer resins and cured after printing [21,22]. The two routes yield different surface textures, degrees of crystallinity and residual-chemistry profiles, and those differences propagate into how each device ages and sheds material in service [23].

The underlying polymer chemistry explains why release differs by route. Polyethylene terephthalate glycol is a semi-crystalline polyester whose ester bonds are susceptible to hydrolysis, so its degradation liberates both particulate fragments and the antimony and tin catalyst residues characteristic of polyester manufacture. Thermoplastic polyurethanes degrade through different linkages and present a distinct additive profile. Methacrylate photopolymers, by contrast, form a cross-linked network whose integrity depends on the completeness of curing, so their principal chemical liability is unreacted monomer and photoinitiator rather than catalyst metals. These chemical distinctions are not academic: they determine which markers a biomonitoring programme should target for each material class, and they explain why a single analytical panel must combine elemental and organic methods [24].

Additives compound the base-polymer chemistry. Aligner and related dental polymers may contain plasticisers, colourants, stabilisers and, in printed resins, photoinitiators, each of which can migrate independently of the polymer backbone and each of which carries its own analytical signature. The additive load is rarely disclosed in full by manufacturers, so an exposure assessment cannot assume a known composition and must instead measure what emerges. This opacity is itself an argument for empirical biomonitoring, because it removes the option of predicting release from a declared formulation [25].

Market scale is part of why the exposure question is pressing. The technology has moved from a niche to a mainstream option within two decades, the market leader, Align Technology, has reported more than twenty-two million cumulative Invisalign patients [7] and directly printed workflows are expanding the manufacturing base further, so even a small per-device release aggregates into a large population exposure. Scale also changes the regulatory calculus, because a material used at this volume in the mouths of children invites the same scrutiny applied to any other high-exposure consumer contact material, and it raises the value of a measurement that could be applied across a large and growing user base.

Mechanical and ageing studies show that both material classes change measurably during laboratory simulations of a treatment course. Water uptake, thermal cycling between the temperatures of food and drink, and cyclic occlusal load together reduce stiffness, roughen surfaces and promote micro-cracking, with directly printed resins showing distinct viscoelastic behaviour and greater moisture sensitivity than thermoformed sheets [23]. Polyester-based materials are additionally prone to hydrolytic and photo-oxidative chain scission, which provides a plausible molecular basis for particle generation and elemental release [24]. These experiments model processes that may occur during wear, but their magnitude and combined action in patients remain to be measured [26].

Clinical use compounds the material question. Aligners are worn for roughly 22 h a day and replaced weekly to fortnightly, so a single patient cycles through dozens of devices during treatment, each contributing a fresh surface to the mouth. Directly printed workflows, prized for chair-side speed and customisation, are expanding rapidly, which shifts part of the device market toward resin chemistry that is sensitive to post-curing [27,28]. The growing use of aligners in children and adolescents changes the exposure calculus further because exposure occurs during development and leaves a longer remaining lifespan in which any retained burden could matter [2]. Table 2 summarises the material and use characteristics that govern release.

## 3. Mechanisms and Evidence of Micro- and Nanoplastic Release

The first direct evidence that aligners shed particles came from a spectroscopic study showing secondary microplastic detachment after seven days in artificial saliva, accompanied by surface changes visible under electron microscopy [29]. That observation reframed aligners from inert appliances into active particle sources and prompted a wave of experimental work that broadened both the conditions tested and the materials compared. The consistent direction of this work is more informative than any single number: release increases with wear time and with mechanical stress, and a substantial fraction of released particles falls below 5 µm, the size range of greatest biological interest [30,31].

Manufacturing route emerges as a decisive variable. Comparative work under simulated oral conditions found that directly printed devices shed more and larger microplastics than thermoformed aligners, with printed particles tending to be more uniform and spherical [31], while thermoformed materials release a greater proportion of nanometre-scale particles under acidic conditions in a single study [32]. The counterintuitive finding that the newer printed technology can release more particulate mass, not less, matters for policy as well as chemistry, because it cautions against assuming that additive manufacturing is inherently cleaner [33]. Released particles are not inert in cell culture, and printed-material particles in particular interact with cells in ways discussed in Section 6 [33].

Three release mechanisms recur across the literature and map onto distinct oral realities. Contact with warm saliva drives hydrolytic degradation and the slow detachment of surface material, a process that intensifies as surfaces roughen. Simulated gastric acid, relevant to swallowed particles, accelerates degradation of polyester-based sheets and couples particle release to elemental release. Cyclic mechanical loading that mimics mastication and daily insertion and removal generates fatigue damage and fresh particles independently of chemical attack [29,32,34]. These pathways may act concurrently in use; single-mechanism experiments therefore may not capture combined release.

Quantitative comparison across studies is hampered by inconsistent metrics. Reports variously express release as particle number, particle mass, mean particle area or count above a size threshold, and they differ in extraction, filtration and counting methods, so a difference between two figures can reflect method rather than material [35]. The strongest claims that survive this heterogeneity are directional: printed devices tend to release more and larger particles, thermoformed materials release more nanoscale particles under acid, and release rises with wear [30,31]. Absolute burdens, by contrast, should be treated as provisional until methods converge. This tension between reliable direction and unreliable magnitude is the central methodological problem of the release literature.

Placing aligner release in the context of other microplastic sources is difficult for the same reason. Dietary and inhalation exposure are estimated in particles or mass per day at the level of whole populations, whereas aligner studies report release from a device under artificial conditions, so the two cannot yet be compared on a common scale. What can be said is qualitative but not trivial: aligner exposure is localised to the mouth and upper gastrointestinal tract, it is continuous for most of the day, and it is delivered by a single identifiable object, which makes it more amenable to source attribution than the diffuse dietary background. Attribution is a genuine advantage of studying a prescribed source, and it is one reason a device-specific biomonitoring signal may be recoverable even against a noisy environmental baseline.

A conceptual distinction sharpens the analysis. Environmental microplastics are largely secondary, formed by the weathering of larger items, whereas aligner-derived particles are generated in situ from an intact device by defined stressors, making them a controlled model of secondary microplastic formation. That control is scientifically useful: the same device, tested under standardised leaching, can serve as a reproducible generator of secondary micro- and nanoplastics for mechanistic and analytical studies, which few real-world sources allow.

Product variability is a further source of scatter that standardisation must confront. Aligner systems differ in polymer grade, sheet thickness, printing resin and post-processing, and even nominally identical products vary between manufacturing batches, so a result obtained for one brand cannot be generalised without care. Rather than treating this as noise to be averaged away, a mature test framework would report release per defined material and process, allowing clinicians and regulators to compare products on a like-for-like basis and creating an incentive for lower-shedding formulations.

Reconciling the two headline findings, that printed devices release more particulate mass while thermoformed devices release more nanoscale particles under acid, resolves an apparent contradiction and carries a practical message. The two material classes present different exposure profiles rather than a simple better-or-worse ranking: a larger mass of micrometre particles from printed devices [31] and a larger number of the most translocation-relevant nanometre particles from thermoformed devices under gastric conditions [32]. A risk assessment based only on mass or only on number could therefore reach different and incomplete conclusions, which is why release should be reported across the full size distribution before any material is judged safer than another.

Wear duration behaves as an exposure variable in its own right. Studies extending contact to about two weeks report rising microplastic release from both material types, with the sub-5 µm fraction increasing over time, which implies that replacement interval may modulate release [30]. Results across multiple commercial brands and both fabrication routes suggest that release is not confined to a single product, although generalisability remains limited by method heterogeneity, product variability and the concentration of key findings in a small number of laboratories [4,5]. Whether individual release studies applied adequate procedural blanks is also uneven, so the contamination scepticism applied to human biomonitoring must apply equally to the release literature. Table 3 collates representative release studies. Table 4 appraises the methodological quality of these studies; procedural blanks are largely unreported and none of the key findings has yet been independently replicated.

**Table 3:** Representative in vitro studies of micro- and nanoplastic release from clear aligners.

Study	Material(s)	Condition	Key Finding
Quinzi et al. 2023 [29]	Thermoformed	Artificial saliva, 7 days	First evidence of secondary microplastic detachment; surface change by microscopy
Orilisi et al. 2026 [30]	Multiple brands	Wear time, composition, process	Release rises with wear time; substantial sub-5 µm fraction
Zecca et al. 2025 [31]	Thermoformed vs. printed	Simulated mastication	Printed devices release more and larger particles
Zecca et al. 2025 [32]	Thermoformed vs. printed	Simulated gastric acid	Acid degradation; Sb/Sn release; nanometre-scale particles reported in one study
Borgese et al. 2026 [33]	3D-printed	Oral simulation, cell exposure	Released particles interact with cells
Barile et al. 2025 [34]	Clear aligners	Cyclic loading	Fatigue damage generates additional particles

**Table 4:** Evidence-quality appraisal of the major in vitro release studies. PET, polyethylene terephthalate(-glycol); PU, polyurethane.

Study	Sample/Materials	Conditions	Particle Identification	Size Detection Range	Blank Control	Replication	Main Limitations
Quinzi et al. 2023 [29]	7 brands (5 PET-based, 2 PU); 2 aligners per group	Artificial saliva, 7 d; stirring 5 h/d (friction)	Raman microspectroscopy + SEM	Mostly 5–20 µm; no nanometre read-out	Not reported	Single laboratory; first report	Short duration; filter-limited size floor; no procedural blanks reported
Orilisi et al. 2026 [30]	10 brands, thermoformed and 3D-printed; 1 pair per group	Artificial saliva, 7 vs. 14 d; 5 h/d stirring; 1.6 µm filtration	ATR-FTIR (material) + Raman + SEM	≥1.6 µm; sub-5 µm fraction rising with time	Not reported	Triplicate runs; same group as [29]	Detection floor excludes nanoplastics; single-laboratory evidence
Zecca et al. 2025 [31]	1 printed (Graphy TC-85) vs. 1 thermoformed (SmartTrack)	Mechanical rubs in ultrapure water	Optical microscopy + TEM + AFM; gravimetry	µm–nm imaged; MP/NP not separable (aggregation)	Laminar-flow handling; blanks not reported	Not replicated	Two products; water, not saliva; aggregation confounds counts
Zecca et al. 2025 [32]	1 thermoformed vs. 1 printed product	Simulated gastric acid (pH 2; 7 d 0.5 M HCl leachates)	AFM (surface) + ICP-MS (elements)	Nanometre surface scale; particles not counted	Acid digestion QC; blanks not reported	Single study; Sb/Sn figures unreplicated	Elemental, not particle, quantification; acid harsher than physiology
Borgese et al. 2026 [33]	Particles generated from 3D-printed aligners (stress + ultrasonication)	Invertebrate model (Hirudo verbana), 24 h–1 wk	Light/electron microscopy; immunoassays; qPCR	MP/NP dispersion; sizes not the endpoint	Controls in bioassay; particle blanks not reported	Triplicate exposures; single laboratory	Invertebrate model; artificially generated particles; dose not wear-relatable
Barile et al. 2025 [34]	3 brands (Essix Ace, Ghost, Invisalign)	Cyclic compressive loading (swallowing simulation)	Optical microscopy + image segmentation	Micrometre range only	Not reported	Not replicated	No chemical (spectroscopic) particle confirmation; risk conclusion exceeds data

## 4. Chemical Leaching: Elemental Residues, Monomers and Additives

Particle release is only one axis of exposure; the same degradation that liberates particles also liberates soluble chemistry, and the two cannot be assessed in isolation. Under simulated gastric conditions, thermoformed aligners co-release antimony at about 0.13 mg/kg, together with tin at roughly 0.09 mg/kg, whereas the directly printed leachate shows a different balance, with less antimony (about 0.03 mg/kg) but slightly more tin (about 0.11 mg/kg) [32]. These figures derive from a single study and require independent replication. Antimony may be useful as a tracer when the polyester composition and background sources are documented, but its detection alone cannot prove aligner origin. Trace-level analytical methods for antimony in PET confirm that the element is measurable in polymer matrices [36].

Antimony trioxide is the polycondensation catalyst most widely used in PET manufacture, and its slow migration from PET into acidic or warm media is well documented in the food-contact literature, which provides useful context for the intra-oral case. The quantities involved are small, and the toxicological significance of chronic low-level antimony exposure from a dental device is unknown, which is precisely why measurement rather than assumption is needed. Framing antimony as a tracer of polyester degradation, rather than asserting a specific toxic effect, keeps the interpretation within what the data support [32,36].

A deeper test of the Sb/Sn proposal is what it would take for these elements to function as valid markers rather than merely detectable ones. Both have appreciable non-aligner backgrounds: antimony reaches saliva through diet, beverages and food in PET packaging and ambient dust, and tin through canned food and other dental materials, so a salivary concentration is uninterpretable without a measured background distribution. ICP-MS also reports total element rather than species, and salivary concentrations are flow- and clearance-dependent (Section 7), so single-time-point values are weak evidence on their own. A defensible marker claim therefore requires, at minimum: documented antimony-catalysed polyester composition of the worn device; a pre-treatment baseline or a matched non-wearer distribution; a within-subject rise across the wear cycle, ideally scaling with wear time; and co-detection of spectroscopically confirmed polymer particles to corroborate origin (Section 8). Until the single-study leachate concentrations are independently replicated and these design conditions are met, Sb and Sn should be treated as candidate markers, suitable for ranking hypotheses and powering study designs, not as established evidence of aligner-derived exposure.

An in vitro leachate concentration is not an ingested dose and cannot be interpreted without measured intra-oral concentrations, exposure duration and background levels. Urethane dimethacrylate has already been quantified in the saliva of appliance wearers, showing that organic leachates can be recovered in vivo [37]. The next step is to establish background distributions and aligner-attributable changes rather than compare a material leachate directly with unrelated regulatory limits.

A point of correction to some early framing is warranted here. Titanium has sometimes been proposed as a marker of aligner degradation, yet that study in fact detected titanium only at low levels in the directly printed resin leachate, plausibly from pigments or fillers rather than from polyester degradation; titanium is in any case not a principal constituent of current aligner materials [32]. Antimony and, where informative tin, are therefore the more defensible elemental markers, and a biomonitoring design that leads with them rests on stronger ground than one built around titanium. Where titanium is detected in the oral cavity it is more plausibly attributable to other dental sources than to aligner shedding, a distinction that any interpretation of salivary titanium must respect [38].

Directly printed resins carry a second, organic chemical liability: incompletely cured monomers and photoinitiators. Post-processing completeness governs the leaching of species such as urethane dimethacrylate, and the concern has now moved from laboratory extraction to patients, with urethane dimethacrylate quantified in the saliva of wearers of printed appliances [25,37]. The bioaccessibility of microplastic-associated additives depends on particle size and on the digestive matrix, so a swallowed particle can deliver a chemical payload that a filtered leachate assay would miss. Work on additive release in simulated human digestion makes this coupling explicit, and studies of artificial digestion show that gastric and intestinal conditions modify both the particle and its leachable load [39,40]. The practical implication is that a credible exposure assessment must measure elemental markers, residual monomers and particles together, because each captures a different facet of the same degrading device. Table 5 summarises the chemical release evidence.

## 5. Particle Size, Morphology and Epithelial Translocation

Size is the central variable that links release to biological plausibility, and it is the variable a biomonitoring method must be built around. Across aligner studies, most quantified particles fall between roughly 5 and 20 µm, but the fine (1–5 µm), submicron (100 nm–1 µm) and strict nanoplastic (<100 nm) fractions are, in that order of increasing plausibility, the sizes most likely to cross oral and gastrointestinal epithelia and reach the circulation [30,31]. The biological relevance therefore concentrates in the tail of the size distribution, not its centre of mass, which has a sobering methodological consequence: the particles that matter most for translocation are the hardest to detect and quantify.

Two anatomical routes are plausible for shed and swallowed particles. The oral mucosa could provide a local path, while the gastrointestinal tract provides a larger absorptive surface for swallowed material. Smaller particles are generally considered more likely to cross biological barriers, but the fraction of aligner-derived material that is absorbed, retained or cleared is unknown [1,41,42]. Modelling internal dose from external release therefore requires the full size distribution, not a single mean, and methods that do not selectively lose the smallest particles during sample preparation.

Morphology adds a further dimension. Printed particles tend to be more uniform and spherical, whereas thermoformed particles are more heterogeneous in shape, and aggregation in any complex fluid can both mask the true number of nanometre particles and alter their apparent bioavailability [31,43]. Uniform spherical particles may be taken up by cells differently from irregular fragments, so shape is not merely descriptive but mechanistically relevant, and it complicates the translation of a laboratory size distribution into a predicted internal dose.

The nanometre fraction poses a detection problem that is qualitatively different from the micrometre one. As particles approach and fall below the diffraction limit, optical spectroscopy loses spatial resolution, aggregation confounds counting, and the mass contributed by each particle becomes vanishingly small, so both particle-number and mass-based methods struggle at once. This is why the size range of greatest biological concern is also the range of greatest analytical difficulty, and why methodological development for nanoplastics, rather than simple application of microplastic methods, is a precondition for meaningful salivary biomonitoring.

In vitro digestion models extend the size-and-fate question beyond the mouth. Sequential oral, gastric and intestinal simulations show that digestion can alter particle surfaces, aggregation and the release of associated chemicals, so the swallowed fraction presented to the gut may differ from the particle shed in the mouth [39,40]. Coupling aligner leaching to such digestion models would provide a more realistic estimate of the material presented to the intestinal epithelium and would connect the intra-oral source to later exposure stages.

Setting aligner exposure against other everyday plastic contacts helps to calibrate concern without dismissing it. A patient is exposed to microplastics from food, water and air regardless of orthodontic treatment, and the aligner increment sits on top of that background rather than replacing it. The increment stands out less for its size than for its character. It is prescribed, localised, chemically identifiable and, in principle, avoidable through material choice. A measured estimate of how the aligner increment compares with the dietary background is exactly the kind of number that would let clinicians and patients reason proportionately, and it is currently absent.

Direct experimental support for translocation-relevant mechanisms is emerging at the oral barrier. Polystyrene nanoplastics compromise epithelial barrier integrity and disorganise tight-junction-related proteins in human buccal epithelial cells, a mechanism by which submicron particles could increase paracellular permeability without overt cytotoxicity [41]. These effects were shown with model polystyrene particles, whose surface chemistry differs from PETG and methacrylate debris, so they establish a mechanistic possibility for the size class rather than a demonstrated effect of aligner-derived material. Particles in the 2 to 10 µm range are taken up by macrophages in vitro, so the released size distribution spans fractions with potentially different biological fates [44]. These observations do not demonstrate systemic uptake from aligners in patients; they define size windows that analytical methods should be able to measure. To keep the evidence classes distinct: the only biological data obtained with actual aligner-derived particles are the macrophage and bioassay responses described in Section 3 and Section 6, whereas all barrier and translocation findings cited here derive from model polymers, chiefly polystyrene. Because surface chemistry, charge and additive load differ among polystyrene, PETG, polyurethane and methacrylate debris, particle size alone is not a sufficient basis for assuming equivalent biological behaviour, and translocation remains an untested hypothesis for each aligner material class separately.

## 6. Biocompatibility and Inflammatory Responses

The biocompatibility literature on aligner materials is larger and older than the microplastics literature, and it is broadly reassuring on acute endpoints while flagging conditional risks. Systematic and umbrella reviews conclude that most aligner materials maintain acceptable short-term cell viability, and that frank cytotoxicity is uncommon with adequately processed devices [45,46]. That reassurance is real but narrow, because acute viability assays are designed to detect cell death rather than the sub-lethal, chronic responses that a years-long exposure might provoke, and because they typically test extracts rather than the particulate fraction.

The conditional risk lies chiefly with directly printed resins. Inadequately post-cured materials can show mild cytotoxicity attributable to residual monomers, and post-process cytotoxicity varies with resin formulation and curing protocol, so a device that is safe when fully cured may not be when it is not [27,28]. This is a manufacturing-quality problem as much as a materials problem, and it reinforces the exposure argument that fabrication route and finishing determine what reaches the patient. It also means that biocompatibility conclusions drawn from ideal laboratory specimens may not transfer to chair-side printed devices with variable curing.

The microplastics perspective adds a mechanism that acute viability assays miss. Aligner-derived particles from printed materials promote M1-like macrophage differentiation and oxidative stress in culture, and live-cell imaging has captured macrophage uptake of these particles [44,47]. If such responses were sustained in vivo, they could contribute to chronic local inflammation, but this has not been demonstrated clinically. Evidence that polyethylene identified in human dental calculus triggers cytotoxic and inflammatory responses in gingival fibroblasts shows that intra-oral polymer particles can be biologically active in vitro, not merely inert debris [48].

Barrier disruption by nanoplastics in buccal epithelium adds a plausible route of increased permeability and potential interaction with co-released chemistry [41]. Read together, the biocompatibility and microplastics literature supports a hypothesis that short-term viability may coexist with sub-cytotoxic inflammatory or barrier effects, especially for small particles and incompletely cured printed materials (Table 6). The hypothesis requires validation under clinically relevant exposure levels and durations; it is not evidence of chronic patient harm.

## 7. Micro- and Nanoplastics in Human Compartments: Associative, Not Causal

The clinical relevance of aligner release depends on whether MNPs reach and persist in the body, and the wider human record answers the reachability question clearly, if not yet the causation question. Particles have been quantified in human blood [18], reported in human placenta and breast milk (as compiled in a recent systematic review of detection studies [42]), and identified in atheromatous plaque, where higher burden was associated with subsequent cardiovascular events [19]. Systematic reviews of human biomonitoring confirm detection across blood, urine and soft tissues, while emphasising heterogeneous methods and the persistent difficulty of relating internal load to any specific health outcome [42,49].

Several routes could carry particles from the oral cavity to internal compartments, but none has been demonstrated for aligner-derived particles in wearers. Swallowed particles may encounter the gastrointestinal epithelium, locally retained particles may interact with oral mucosa, and inhaled environmental particles provide a separate route unrelated to the device. Wider human detections establish internal presence but not the source or pathway [1,42,49].

Two cautions must travel with these data at all times. The first is that the evidence is associative: detection, and even correlation with outcomes, does not establish that MNPs cause disease, still less that aligners specifically contribute to any measured burden [1,19]. Responsible communication of this field depends on holding that line, because the gap between plausible harm and demonstrated harm is exactly where public understanding is most easily distorted. The second caution is that methodological heterogeneity and contamination risk mean some reported burdens are uncertain, which is an argument for standardisation and blank control rather than for dismissal of the signal [42].

Moving from association towards causation will require the classic ingredients of causal inference: a measured internal dose, a plausible and demonstrated mechanism, a dose–response relationship, and temporality in which exposure precedes outcome. Aligner wear is well suited to supply several of these, because the exposure has a defined start, a graded intensity through wear time and material choice, and an identifiable population, which together support a dose–response design that ambient exposure cannot easily provide. The missing ingredient is the measured internal dose, which returns the argument to biomonitoring as the rate-limiting step for the whole causal question.

Biomarker kinetics are an under-examined part of the design. A salivary measurement captures a moment in a dynamic system in which particles are continuously shed, cleared by swallowing and salivary flow, and partly retained, so the relationship between an instantaneous salivary concentration and cumulative internal dose is not straightforward. Interpreting a salivary marker will require at least a basic understanding of shedding and clearance rates, ideally from repeated sampling across a wear cycle, so that a single measurement can be placed on a time course rather than read in isolation.

A rudimentary compartmental model makes this tractable and shows how a time series of salivary concentrations can be converted into a source-strength estimate. Let R(t) be the release rate of an aligner-derived analyte into a well-mixed salivary pool of resident volume V, cleared by swallowing and salivary flow Q as a first-order process with rate constant k ≈ Q/V; then dS/dt = R(t) − kS(t), where S is the analyte mass in the pool and the measured concentration is C(t) = S(t)/V. Because salivary clearance half-times (minutes) are short relative to changes in shedding (hours to days), the pool is effectively at quasi-steady state, so C(t) tracks R(t) almost instantaneously and the source strength follows directly as R(t) ≈ Q·C(t). Cumulative release over a wear interval is then the integral of Q·C(t) dt, estimated by trapezoidal integration of repeated concentration measurements multiplied by the participant’s measured salivary flow—which is why the proposed sampling protocolrequires flow-rate-adjusted reporting and repeated within-subject sampling. The model’s simplifications are explicit: a well-mixed single pool, first-order clearance, no mucosal or device re-adsorption and analyte stability; retention makes Q·C(t) a lower bound on release, and a two-compartment extension with a retained fraction can be fitted once repeated-sampling data exist. Even in this rudimentary form, the model converts the proposed biomonitoring from signal detection into quantification, because every parameter it needs—C(t), Q and the sampling times—is measured by the sampling protocol proposed in Section 8.

No direct aligner-derived exposure data were identified for patients with cleft lip and palate. Their prolonged, appliance-intensive care from early life makes them a candidate subgroup for future study, but this is a research hypothesis rather than established evidence. Pregnancy is a separate potential priority because wider human studies report placental detections, again without source attribution. These considerations identify cohorts in which measured exposure data could be especially informative; they do not justify withholding clinically indicated care.

Children warrant separate consideration. Intake relative to body mass is higher, detoxification systems are less mature, and developmental windows are sensitive, so an equivalent external exposure translates into a larger internal dose and a longer horizon over which effects could manifest. Early assessments of nanoplastic exposure in children make the paediatric question concrete rather than hypothetical, and they justify prioritising children in any biomonitoring programme built around a prescribed exposure such as aligner wear [20]. The convergence of a reachable internal compartment, an unmeasured prescribed source and a vulnerable and growing user group is what elevates aligner MNPs from a materials curiosity to an exposure-science priority. Table 7 summarises the human compartment evidence and its interpretive limits.

## 8. An Analytical Toolbox for Salivary Biomonitoring

Saliva is the priority first-line matrix for source-proximal aligner biomonitoring. It is in continuous contact with the device and can be collected without needles and with minimal burden [50]. A salivary signal would index local release and oral exposure, not a systemically absorbed dose, so it is a starting point from which internal uptake could later be investigated. Two analytical tasks follow: elemental markers of polymer degradation must be quantified at trace levels, and released particles must be identified and sized across a range extending toward the nanometre regime. The tasks are complementary and should be run in parallel.

For the elemental task, ICP-MS provides high sensitivity, but saliva-specific limits of detection and quantification for antimony and tin must be established during validation [51,52]. Collision or reaction cell technology can reduce spectral interferences, and closed-vessel digestion can limit loss and contamination [52]. Methods for titanium, vanadium and zirconium in oral mucosa demonstrate the feasibility of trace-element analysis in oral biological samples, but they do not validate antimony or tin measurement in saliva [38].

Particle identification and sizing rely on vibrational spectroscopy and electron microscopy, which are complementary rather than interchangeable. Routine Raman microspectroscopy commonly resolves particles around 1 µm, with submicron analysis requiring specialised configurations and careful control of fluorescence; micro-FTIR is faster but commonly underestimates particles below about 20 µm [43,53]. ATR-FTIR can extend usable detection to roughly 6 µm in suitable samples [54]. Surface-enhanced Raman approaches are promising for nanoplastic detection but are not yet validated for aligner-derived particles in saliva [55,56]. Electron microscopy supplies morphology and nanoscale sizing but lacks stand-alone polymer specificity and requires artefact-aware sample preparation [57].

The decisive obstacles in saliva are not raw sensitivity but control of the matrix and of contamination. Saliva is protein-rich and variable in viscosity, so digestion, filtration or micro-extraction is needed before instrumental analysis, and in vivo solid-phase micro-extraction has already been validated for recovering trace contaminants directly from human saliva, which points to a practical clean-up route [58]. Procedural contamination is the dominant threat to any MNP measurement, because plastic is everywhere in the laboratory, and blank-driven quality control has moved from good practice to a non-negotiable requirement [59]. A further gap is the absence of saliva-specific certified reference materials, so validation currently borrows from hair, urine and water reference materials with spike-recovery confirmation, an interim solution that standardisation efforts must replace [60].

No single technique answers every question, so a tiered strategy is the realistic design. Spectroscopic and microscopic methods identify and size individual particles but are slow and struggle below the resolution limit, whereas mass-based approaches such as pyrolysis coupled to gas chromatography-mass spectrometry quantify total polymer mass without particle counts, and the two are complementary, not competing. A defensible salivary workflow would screen for elemental markers by ICP-MS, confirm and size particles by Raman and electron microscopy on a subset, and cross-check polymer mass where sample volume allows, with every stage carried under blank-controlled conditions. Building this tiered logic into the protocol from the outset avoids the false economy of choosing one method and discovering its blind spot later.

Stated critically, these limits mean that the size fractions of greatest biological concern are at present the least measurable in saliva: routine Raman resolves particles only down to about 1 µm, micro-FTIR underdetects well above that bound, electron microscopy sizes nanoparticles but cannot alone confirm polymer identity, surface-enhanced Raman and mass-based pyrolysis approaches are not yet validated in saliva, and no saliva-specific reference materials exist against which any of them could be qualified. A realistic programme must therefore be staged: what is achievable now is blank-controlled quantification of elemental markers and of particles larger than about 1 µm, while the submicron and strict nanoplastic tiers remain a method-development target rather than a measurable endpoint. Interim salivary results should state this censoring explicitly, so that an absence of detected nanoplastics is read as an instrument limit, not as evidence of absence.

Protocol detail determines whether results are comparable. Artificial-saliva formulations should report pH, ionic strength and relevant macromolecular components because these variables can affect hydrolysis and particle aggregation; simplified formulations may produce different release profiles. Thermal and mechanical cycling should likewise be reported in enough detail to reproduce the temperature range, dwell time, load, frequency and duration. Standardising these parameters would allow laboratories to compare release from the same material under the same defined conditions.

Contamination control in saliva work deserves particular emphasis because the matrix is collected, handled and processed in a plastic-rich clinical environment. Collection tubes, pipette tips, filters and airborne fibres can add particles indistinguishable from the analyte, so field blanks, procedural blanks and positive controls must accompany every batch, and the collection device itself must be screened for the polymers of interest. Without this discipline a salivary microplastic measurement is difficult to interpret, and part of the variability in human biomonitoring may reflect contamination as well as biological variation [59].

Transfer of methods from blood and urine to saliva is feasible but not automatic. Blood and urine protocols provide validated templates for digestion, extraction and trace-element quantification, yet saliva differs in protein content, viscosity and the diurnal variability of its composition, so calibration and clean-up must be re-optimised rather than imported unchanged. The pay-off for solving these matrix problems is substantial, because a validated salivary method would replace an invasive blood draw with a sample a patient can provide in the dental chair, which is precisely what makes population-scale biomonitoring of a prescribed exposure imaginable [50].

Emerging sensing platforms point towards the eventual field stage of such a programme. Electrochemical and optical biosensors for salivary analytes, microfluidic point-of-care devices and wearable formats are advancing quickly, and although none is yet validated for aligner-derived micro- and nanoplastics, they define a plausible route from centralised reference methods to chair-side screening [61]. The sensible sequence is to establish reference measurements first and to adapt these platforms afterwards, so that a convenient device is calibrated against a trusted method rather than deployed ahead of one. Treating point-of-care sensing as the endpoint of validation, not its substitute, keeps enthusiasm for new hardware from outrunning the underlying measurement science.

Quality assurance across laboratories is the connective tissue that turns individual methods into a field. Inter-laboratory comparison, shared blinded samples and agreed reporting minima for blanks, recovery and limits of detection are what allow results from different groups to be pooled with confidence, and their absence is a large part of why the current human microplastics literature is difficult to synthesise. Building these practices into aligner biomonitoring from the start, rather than retrofitting them, would let the field avoid the reproducibility problems that have slowed the wider microplastics community, and would make the eventual dataset usable for regulatory as well as academic purposes, avoiding the standardisation gap that has been flagged more broadly across plastics research [62].

Standardised collection is as important as instrumental sensitivity, and it is easy to neglect. Salivary analyte concentrations vary with time of day, recent food and drink and collection device, so a biomonitoring protocol must fix these variables, specify a collection matrix that does not itself shed plastic, and control for them in children who may not tolerate timed unstimulated collection [63]. Data analysis is a further frontier: chemometric and machine-learning classification of spectra improves throughput and consistency, and it is increasingly integral to nanoscale particle identification rather than an optional add-on [55]. Point-of-care microfluidic and microsampling platforms hint at eventual field deployment and at biomonitoring that could scale to whole cohorts, but standardised protocols and reference materials remain the prerequisite for any of it [61,64]. Table 8 sets out the toolbox and its readiness. Table 9 condenses these requirements into a specimen-level sampling and analysis protocol for the first wearer studies.

**Table 8:** Analytical methods for salivary biomonitoring of aligner-derived exposure.

Method	Target	Detection/Resolution	Main Control Need	Evidence
ICP-MS	Sb, Sn (Ti excluded)	Method-dependent trace levels; saliva-specific LOD/LOQ not established	Digestion, matrix-matched calibration	[51,52]
Raman microspectroscopy	Particle ID	About 1 µm in routine use; submicron requires specialised setups	Fluorescence mitigation	[55,56]
Micro-FTIR (incl. ATR)	Particle ID	~20 µm (ATR to ~6 µm)	Small-particle underestimation	[53,54]
SEM/TEM	Morphology, sizing	Nanometre scale	Sample prep, contamination	[57]
Saliva micro-extraction	Sample clean-up	Recovery of trace analytes	Protein removal	[58]
QA/QC and CRMs	Validation	Blank-limited	Saliva-specific CRMs lacking	[59,60]

**Table 9:** Proposed specimen-level salivary sampling and analysis protocol for aligner wearers. Saliva indexes local, source-proximal exposure, not systemic dose. LOD/LOQ, limits of detection/quantification.

Protocol Element	Proposed Specification
Saliva type	Unstimulated whole saliva as the primary matrix; stimulated collection only as a documented fallback in young children, analysed separately
Sampling timing	Standardised morning window; ≥60 min after food, drink or oral hygiene; paired samples immediately before insertion of a fresh aligner and after a defined wear interval (e.g., end of week 1); time since last insertion recorded
Sample volume	≥2 mL per aliquot, with separate aliquots for elemental (ICP-MS) and particle (Raman/electron microscopy) workflows
Collection	Passive drool into pre-screened glass or certified low-particle vessels; no cotton or absorbent collection devices (fibre shedding, analyte sorption)
Pre-treatment and storage	Immediate cooling; storage at −20 °C or below; freeze–thaw cycles limited and logged; acidification of elemental aliquots after subsampling
Digestion/filtration	Elemental: closed-vessel acid digestion. Particles: enzymatic or alkaline matrix digestion, filtration onto pre-cleaned filters; filter pore size reported as the explicit lower size cut-off
Blanks	Field blanks opened at the collection site, procedural blanks in every batch, and collection-device blanks; LOD/LOQ derived from the blank distribution
Recovery	Spike–recovery for Sb and Sn at three levels in pooled saliva; particle recovery with characterised reference particles (e.g., PET microspheres); pre-defined acceptance criteria
Normalisation	Concentrations reported per volume with flow-rate-adjusted values; collection duration recorded; salivary protein or osmolality as covariates
Repeated-measures design	Within-subject sampling: pre-treatment baseline, then fixed intervals across the wear cycle, so each participant serves as their own control
Controls	Matched non-wearer controls; diet documented and full intra-oral polymer device inventory recorded for all participants

## 9. Aligners Among Intra-Oral Polymer Sources and Exposure in Context

Aligners do not sit alone in the mouth. Retainers, occlusal splints, mandibular advancement devices, resin-based composites, adhesives and prosthetic bases are all polymer sources subject to the same degradation chemistry, and several have been flagged as hidden contributors to intra-oral microplastic and monomer exposure [3]. Treating aligners as an isolated problem risks both over-attributing a salivary signal to them and missing the cumulative burden a patient carries from several coexisting devices. A biomonitoring design must therefore record the full intra-oral polymer inventory of each participant, so that aligner-specific release can be separated from the shared background.

Practical source discrimination is nonetheless feasible, because the candidate sources differ chemically, and five strategies can be combined. First, polymer-specific spectroscopy: Raman and FTIR spectra distinguish PETG and polyurethane aligner debris from the methacrylate signatures of composites, adhesives and prosthetic bases. Second, chemical fingerprints: elemental ratios (Sb/Sn from polyester catalysis) and organic leachates (urethane dimethacrylate from printed resins) tied to the documented composition of the worn device. Third, pre/post-treatment longitudinal sampling, in which each patient’s pre-insertion baseline captures their personal dietary, packaging and airborne background. Fourth, within-treatment temporal structure: sampling across the replacement cycle, since aligner-derived release should track fresh-device insertion whereas background sources should not. Fifth, control populations matched for the other intra-oral polymers, including retainer-only wearers. None of these is individually decisive, but their agreement—an aligner-typical polymer spectrum, a composition-consistent chemical fingerprint and a baseline-referenced, wear-locked temporal pattern absent in controls—would constitute strong source attribution (Table 9).

The comparison also clarifies why aligners are a useful sentinel despite this complexity. Unlike a permanent restoration, an aligner is worn on a clinician-set schedule and replaced frequently, so its exposure has a defined temporal structure. Evidence that polymer particles can be biologically active, including macrophage responses and inflammation associated with polyethylene detected in dental calculus, supports general biological plausibility but not direct equivalence across device classes [48]. Salivary monomer measurements in appliance wearers show that an analytical signal is recoverable for at least one chemical endpoint, supporting extension to a broader exposure panel [25,37].

What distinguishes the intra-oral route is not its mass but its structure: continuous delivery to a defined mucosal surface from a single, chemically identifiable and controllable source. That lets a measured burden be attributed to a specific device and, in principle, tested by changing the device, an experimental control few environmental exposures offer, and the deeper reason aligners deserve a dedicated measurement effort.

## 10. Regulatory Context, Exposure Compartments and Safe-and-Sustainable-by-Design Framework

Aligners embody a One Health tension because each appliance is a prolonged personal exposure and a short-lived, single-patient plastic product, and both issues originate in the material and manufacturing process [3,5]. A treatment course discards dozens of thin polymer shells, producing a material-flow problem that mirrors the environmental burden studied by the wider microplastics field. Personal and environmental exposure are therefore two stages in the life of the same object.

The waste dimension deserves more attention than it usually receives. Used aligners are thin, sometimes multi-material and contaminated with saliva, and dedicated recycling routes are not established in the evidence reviewed here. End-of-life handling is therefore likely to depend on local clinical or household waste systems, but quantitative data are sparse. A treatment episode generating many devices per patient, multiplied across a growing market, creates a material stream whose scale and fate require direct measurement rather than assumption.

Mitigation options exist on both sides of the loop and reward measurement. On the material side, lower-shedding formulations, more complete curing of printed resins and bio-based or more stable polymers could reduce release at source, but their benefit can only be demonstrated against a validated release metric. On the waste side, take-back schemes, material recovery and design for recyclability could shrink the environmental stream, though the multi-material, saliva-contaminated nature of used aligners makes this genuinely difficult. Measurement underpins both routes, because neither a cleaner material nor a recyclability claim can be credited without a method to verify it.

Regulatory change in Europe sharpens the broader substitution question without directly driving aligner use. Regulation (EU) 2024/1849 [8], amending Regulation (EU) 2017/852 on mercury [9], phases out routine dental amalgam use across the Union from 1 January 2025, subject to stated derogations. Its direct practical effect concerns restorative dentistry, not orthodontic aligners. In parallel, polymer-based appliances and 3D-printed devices are expanding for independent clinical and manufacturing reasons. REACH restriction (EU) 2023/2055 [10] addresses intentionally added synthetic polymer microparticles, while Regulation (EU) 2017/745 [11] governs medical device conformity, risk management and post-market surveillance. The gap is precise: wear-generated particles are not the same as intentionally added microparticles, and no harmonised dental-specific regime combines long-term chemical migration, particle generation, biomonitoring and circularity in one test package [5].

To be precise about the relationship: dental amalgam is being replaced by polymer restoratives, not by aligners, and no regulatory or clinical substitution links the two device classes. The amalgam phase-out matters here for a different reason. It illustrates a general principle of materials policy: a material removed on the strength of a characterised hazard and measured release should not be succeeded by materials whose own release remains unmeasured. Applied to the expanding polymer device class as a whole, aligners included, this is a safe-and-sustainable-by-design argument rather than a substitution claim—the lesson of amalgam is the evidentiary standard it was held to, and the proportionate response is to hold expanding polymer devices to a comparable standard of release measurement before their use grows further. The resolution is not to reverse amalgam phase-out, which rests on solid environmental grounds, but to measure what polymer devices actually release. Reviews of the aligner field note that policy discussion is already running ahead of the exposure data needed to inform it, which is precisely the condition under which measured biomonitoring adds most value [5].

Exposure may also extend beyond the individual patient. Dental staff may encounter particles during trimming, finishing, polishing and appliance adjustment, but direct monitoring data for aligner-related occupational exposure are sparse [65]. Environmentally, plausible pathways include chairside dust, wastewater transport of fine debris and disposal of serial single-patient appliances. Screening frameworks can support contamination-aware identification of suspect particles in human tissue, but they do not attribute those particles to a dental source [66]. Life-cycle comparisons for aligner therapy remain limited, and the environmental arm of the exposure question is therefore as poorly quantified as the clinical one.

**Figure 2:**
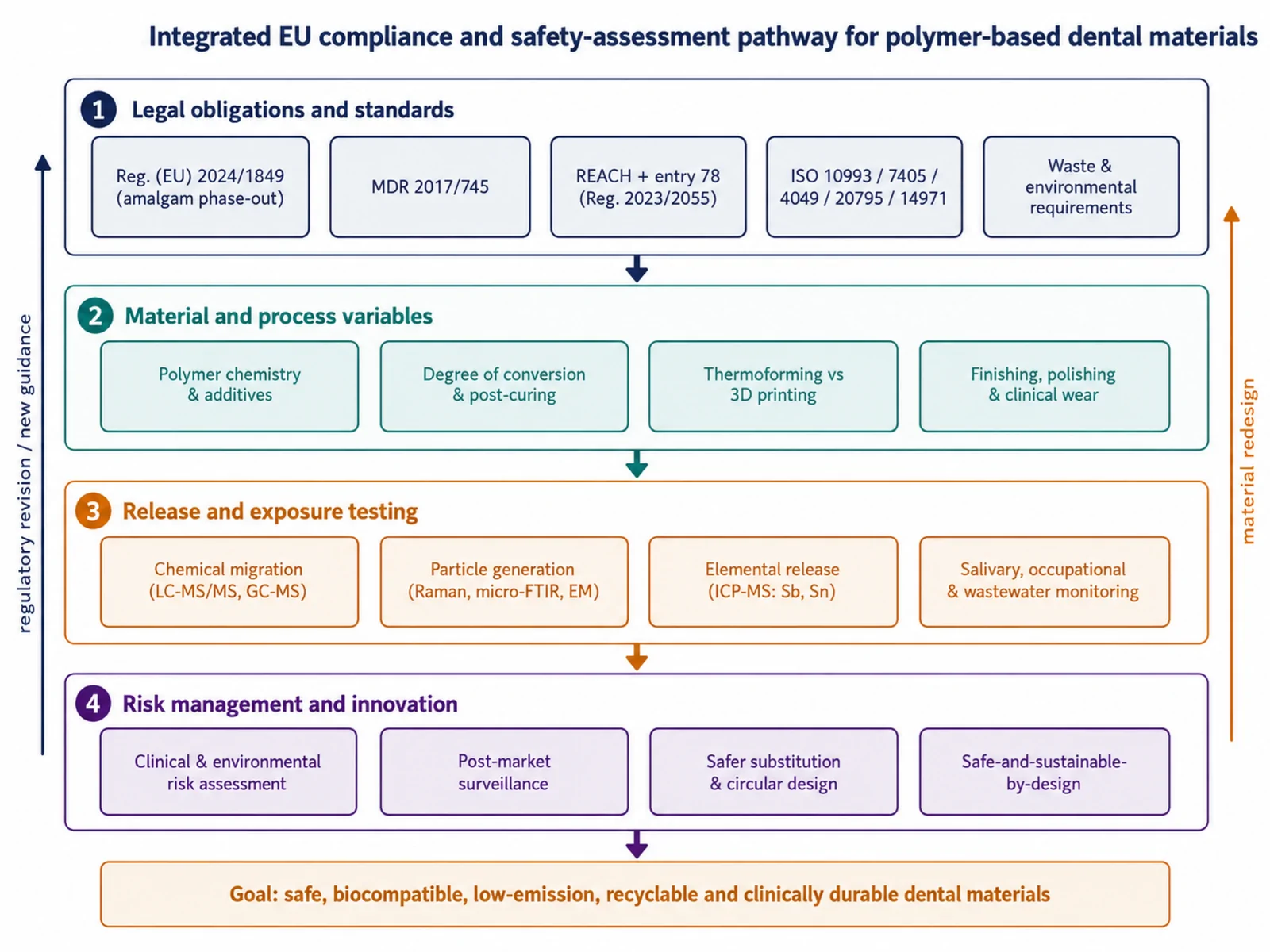
Integrated EU compliance and safety-assessment pathway for polymer-based dental materials. The legal instruments shown are Regulation (EU) 2024/1849 [8], Regulation (EU) 2017/745 [11] and Commission Regulation (EU) 2023/2055 [10]; the standards shown are ISO 10993-1 [12], ISO 10993-18 [13], ISO 7405 [14], ISO 4049 [15], ISO 20795-2 [16] and ISO 14971 [17]. The amalgam phase-out is shown as a broader substitution context, not a direct legal driver of aligner use or wear-generated particle regulation. Feedback from testing, post-market evidence and environmental monitoring informs material redesign and regulatory guidance.

The proportionate response is to widen the compliance target from short-term biocompatibility towards release-based testing across the product life. Safe-and-sustainable-by-design offers a workable frame: a high degree of conversion and optimised post-curing to limit residual monomer, lower-leaching and lower-shedding formulations, reduced endocrine-active impurities, recyclable or reusable formats where clinically feasible and post-market surveillance of release-related exposure. These levers are experimentally tractable: printable formulations for removable orthodontic appliances have been modified and evaluated for post-cure-dependent cytotoxicity and for release into artificial saliva at two pH values over 42 days, though without accompanying measurement of particle shedding [67]. Figure 2 sets this out as an integrated pathway that links legal obligations to controllable material and process variables, then to release and exposure testing, and finally to risk management and product redesign, with feedback from post-market and environmental evidence back into both the material and the rule. Under that model the amalgam phase-out is a first step in modernising dental-material safety rather than an endpoint.

The comparison with amalgam is instructive rather than rhetorical. Amalgam was regulated on the strength of a characterised mercury hazard and a measurable environmental release, which is precisely the evidence base that polymer alternatives lack. A mature materials policy would demand comparable exposure data for any expanding successor material, so that a decision taken for sound environmental reasons is not undercut by an unmeasured health-and-environment trade-off. For aligners specifically, the proportionate step is to quantify release and let the evidence shape both clinical choice and regulation.

## 11. Evidence Gaps and a Research Roadmap

Four gaps recur across the evidence assembled here. There are no in vivo human data on aligner-derived MNP burden and no salivary biomonitoring in wearers; nanometre-scale quantification in biological matrices remains difficult and method-dependent; long-term and cumulative effects, especially in children, are unknown; and analytical protocols are not standardised, so studies are suggestive rather than comparable [4,5]. That first gap is narrower than it appears: metal-ion release from fixed orthodontic appliances has been examined in vivo in an animal model [68], but the equivalent measurement has never been attempted for aligner-derived micro- and nanoplastics, in animals or in wearers. These gaps are addressable, and orthodontics is unusually well placed to address them, because the device, the dose schedule and the exposed population are all defined in advance, which removes much of the uncertainty that hampers ambient-exposure studies.

We propose two concrete and sequenced steps, summarised with the underlying exposure logic in Figure 1. The first is a harmonised in vitro leaching assay that defines artificial-saliva composition, thermal and mechanical cycling, wear interval and validated read-outs across the sub-5 µm and nanometre ranges, with mandatory procedural blanks, so that results from different laboratories become comparable rather than merely directionally consistent [30,59]. Standardisation of this kind is the single change that would most improve the release literature, because it would convert a scattered set of positive findings into a quantitative, poolable evidence base, and it would allow release to be expressed in a common currency of number and mass per unit area per unit time.

The second step is validated non-invasive salivary biomonitoring in wearers, pairing ICP-MS for antimony and tin with vibrational spectroscopy and electron microscopy for particles, embedding blank-driven contamination control throughout, and prioritising children in whom exposure may begin early and continue for months or years [26,64]. Development of saliva-specific certified reference materials should proceed in parallel, since validation currently leans on materials designed for other matrices. A staged programme is realistic: first standardise the leaching assay and its read-outs, then transfer the validated methods to a small paediatric and adult wearer cohort with matched non-wearer controls and only then scale to a larger longitudinal study capable of relating salivary burden to time in treatment. Point-of-care platforms could support the later stages once the reference methods are fixed [61].

Cohort design in children raises specific requirements. Timed, unstimulated saliva collection may be difficult in young participants, so age-appropriate devices and protocols are needed, alongside careful control of confounders such as diet, other intra-oral polymers and time since eating. Ethical design must weigh the value of early exposure data against the burden on child participants and secure genuine assent as well as parental consent, favouring the least invasive sampling compatible with a reliable signal. A matched non-wearer control group is essential, because a salivary marker means little without a baseline against which aligner-attributable release can be judged.

Translating findings responsibly matters as much as generating them. Clinicians and patients will reasonably ask what a measured salivary burden means for health, and the honest answer for now is that it quantifies exposure, not harm. Communicating that distinction clearly, without either dismissing the finding or inflating it into a health scare, is part of the research obligation, and it argues for involving orthodontists, exposure scientists and patient representatives in study design from the outset. Standards bodies and funders have a role in convening the harmonisation that no single laboratory can impose, and their early involvement would shorten the path from scattered in vitro reports to a validated, comparable measurement.

The scale of this programme suits multi-site collaboration rather than single-laboratory work. A harmonised leaching protocol and a multi-site biomonitoring cohort, with shared reference materials and blinded inter-laboratory comparison, fit the model of coordinated environmental-health and circular-economy research and the remit of standards organisations. Treating aligner exposure as a well-bounded test case, rather than one more entry in the broad microplastics literature, would help deliver the first patient-level exposure estimates within a realistic horizon.

This review has limits that shape those recommendations. It is narrative rather than systematic, so it does not quantify pooled effect sizes, and it rests on a release literature that is young, heterogeneous and dominated by in vitro work, so its conclusions about magnitude are provisional. Four constraints deserve explicit acknowledgement: much of the key evidence, including the antimony figure and the nanoscale-in-acid finding, comes from a small number of laboratories without independent replication; no validated method has yet quantified aligner-derived micro- or nanoplastics in saliva of wearers, so the particle-biomonitoring proposal is a development target rather than a ready tool; the barrier and translocation mechanisms rest partly on model polystyrene particles rather than aligner polymers; and no estimate exists of how the aligner dose compares in magnitude with dietary or airborne exposure. What the evidence supports is qualitative and directional: release occurs in vitro, depends on size and test conditions, is chemically as well as physically complex, and has not been quantified as a particle burden in patients. Those conclusions justify method development without overstating current knowledge.

## 12. Conclusions and Future Directions

Clear aligners are a prescribed and expanding source of particle and elemental release under laboratory conditions, but aligner-derived particle burden and systemic dose in wearers remain unmeasured [30,31,32]. The available in vitro evidence, concentrated in a small number of laboratories, indicates that release depends on manufacturing route and test conditions, with directly printed devices tending to shed more micrometre-scale material and polyester-based thermoformed materials releasing Sb/Sn under simulated gastric conditions [30,31,32]. Wider human studies show that micro- and nanoplastics can be detected in internal compartments, but those findings are not source-specific and do not establish causation [18,19,42,49]. With respect to chronic particle-related risk from aligners, the evidence is insufficient to establish either safety or harm. Salivary ICP-MS for Sb and Sn is technically plausible but requires saliva-specific validation, whereas quantification of the submicron particle fraction remains a method-development target [51,52,53,54,55,56,57].

Future directions follow directly from these gaps. Harmonised in vitro release testing and validated salivary biomonitoring, with age-appropriate cohorts and strict contamination control, would replace assumption with measured source-proximal exposure and build the basis needed to investigate systemic uptake and health outcomes [59,63,64]. Priorities are as follows: (i) a standardised leaching assay with validated sub-5 µm and nanometre read-outs and mandatory blanks; (ii) saliva-specific certified reference materials and ICP-MS validation for antimony and tin; (iii) staged wearer cohorts, prioritising children, with matched non-wearer controls; and (iv) integration of release-based testing into safe-and-sustainable-by-design principles and post-market surveillance for polymer dental devices. Pursuing these steps before aligner use expands further would convert a currently unmeasured prescribed exposure into a quantified, regulatable one.

## Figures and Tables

**Table 1:** The five-stage exposure pathway for aligner-derived micro- and nanoplastics: definitions, measurement routes, and evidence currently available. MNP, micro- and nanoplastics; Sb, antimony; Sn, tin.

Stage	Definition	How It Can Be Measured	Evidence Currently Available
1. Device release	Particle and chemical release from the device under oral-, gastric- or load-simulating conditions	In vitro leaching with microscopy, vibrational spectroscopy and ICP-MS	Demonstrated in vitro (Section 3); direction consistent, magnitudes method-dependent
2. Salivary concentration	Aligner-derived particles and elemental/organic markers in wearer saliva (source-proximal external exposure)	Validated salivary ICP-MS (Sb/Sn) with Raman and electron microscopy on particles (Section 8)	Not yet measured for particles; urethane dimethacrylate quantified in wearers’ saliva (Section 4)
3. Swallowed/oral-contact dose	Daily amount presented to the gastrointestinal tract and oral mucosa	Salivary flow and clearance modelling; simulated digestion coupled to leaching (Section 5)	Not measured; digestion models show particles and their leachable load are modified in transit (Section 5)
4. Epithelial uptake	Translocation across oral or gastrointestinal epithelium (internal exposure)	In vitro barrier and uptake models; future ex vivo and animal studies	Mechanistic only, largely from model particles and in vitro macrophage uptake (Section 5 and Section 6); not demonstrated for aligner particles in patients
5. Systemic burden	Particles or markers retained in blood and tissues (internal dose)	Blood and tissue MNP analytics under strict QA/QC (Section 7)	Human detections exist (Section 7) but are not source-specific; no aligner attribution

**Table 2:** Characteristics of clear aligner types relevant to micro- and nanoplastic exposure.

Feature	Thermoformed Aligners	Directly 3D-Printed Aligners	Relevance to Exposure
Base polymers	PETG, thermoplastic polyurethane	Methacrylate photopolymer resins	Determines degradation chemistry and elemental residues [21,22]
Fabrication	Heat-pressing of a sheet over a model	Additive printing plus post-curing	Post-curing completeness governs monomer leaching [27,28]
Ageing behaviour	Hydrolysis, surface roughening	Moisture uptake, viscoelastic change	Both roughen and shed with service time [23,24]
Typical wear	~22 h/day, replaced weekly to fortnightly	~22 h/day, replaced weekly to fortnightly	Prolonged, repeated intra-oral contact [26]
Population	Adults and, increasingly, children	Adults and, increasingly, children	Growing paediatric use motivates age-specific exposure research [2]

**Table 5:** Chemical species released from clear aligners and related intra-oral polymers.

Species	Source/Route	Reported Behaviour	Evidence
Antimony (Sb)	PET catalyst residue	~0.13 mg/kg under gastric acid (single in vitro study, not yet replicated); possible tracer only when composition and background are known	[32,36]
Tin (Sn)	Catalyst residue	Released by both material classes under acid (printed leachate slightly higher, ~0.11 vs. ~0.09 mg/kg); not material-discriminating on its own	[32]
Titanium (Ti)	Proposed marker; other dental sources	Detected only at low levels in printed-resin leachate (pigment/filler origin); not a polyester-degradation marker; other intra-oral sources more likely	[32,38]
Urethane dimethacrylate	Uncured monomer, printed resin	Detected in patient saliva; depends on post-curing	[25,37]
Microplastic additives	Leached from swallowed particles	Bioaccessibility depends on size and digestion	[39,40]

**Table 6:** Biocompatibility and inflammatory findings for aligner materials and intra-oral polymer particles.

Model/Endpoint	Material	Finding	Evidence
Systematic/umbrella reviews	Mixed	Generally acceptable short-term viability	[45,46]
Post-process cytotoxicity	Printed resin	Mild toxicity if under-cured	[27,28]
Macrophages	Printed particles	M1-like activation, oxidative stress, uptake	[44]
Fibroblasts, keratinocytes	3D-printed devices	Inflammatory response to particles	[47]
Buccal epithelium (TR146)	Nanoplastics	Barrier disruption, tight-junction change	[41]
Gingival fibroblasts	Polyethylene in calculus	Cytotoxic and inflammatory response	[48]

**Table 7:** Evidence for micro- and nanoplastics in human compartments and its interpretation.

Compartment	Evidence	Association Reported	Interpretive Limit
Blood	Quantified in multiple studies	Systemic circulation	Contamination control varies [18,49]
Atheroma	Detected in plaque	Cardiovascular events	Associative, not causal [19]
Placenta, breast milk	Detected	Early-life exposure	Detection only [42]
Soft tissues, urine	Detected	Systemic distribution	Method heterogeneity [49]
Children	Emerging salivary exposure data; no aligner-specific particle data	Heightened vulnerability	Few quantitative studies [20]

## Data Availability

No new data were created or analysed in this study. Data sharing is not applicable to this article; all evidence derives from the cited published sources and legal instruments.
